# Increased risk of smoking and pain in individuals with intermittent explosive disorder in the All of Us dataset

**DOI:** 10.3389/fpsyt.2025.1695905

**Published:** 2025-12-11

**Authors:** Fiona Ralston, Michael L. Thomas, Murray B. Stein, Emil F. Coccaro, Alejandro D. Meruelo

**Affiliations:** 1 Department of Psychiatry, University of California San Diego, San Diego, CA, United States; 2 Department of Psychology, Colorado State University, Fort Collins, CO, United States; 3 Psychiatry Service, VA San Diego Healthcare System, San Diego, CA, United States; 4 Department of Psychiatry and Behavioral Health, The Ohio State University Wexner Medical Center, Columbus, OH, United States

**Keywords:** pain, tobacco, major depression, generalized anxiety (GAD), intermittent explosive disorder, propensity score matching (PSM)

## Abstract

**Introduction:**

Intermittent Explosive Disorder (IED) is characterized by recurrent impulsive aggression and has been linked to impairments in physical and psychological functioning. However, population-based evidence examining health-related correlates of IED remains limited. This study evaluated whether individuals with IED differ from matched controls in tobacco use and pain, two clinically relevant health domains.

**Methods:**

Data were drawn from the All of Us Research Program. Individuals with IED were matched 10:1 to participants without IED based on age, sex, race/ethnicity, and income. Group differences in tobacco use and pain were examined using chi-square analyses and logistic regression. Models estimated odds ratios (ORs) with 95% confidence intervals (CIs), with multivariable models additionally adjusting for major depressive disorder (MDD) and generalized anxiety disorder (GAD).

**Results:**

Relative to matched controls, individuals with IED showed significantly higher odds of lifetime cigarette smoking, and this association remained robust after adjusting for MDD and GAD. Participants with IED also demonstrated significantly greater odds of reporting moderate to severe pain during the past seven days.

**Discussion:**

Findings suggest that IED is associated with elevated vulnerability to both smoking and pain, independent of co-occurring depressive and anxiety disorders. These results highlight the importance of integrating tobacco cessation and pain management strategies into clinical care for individuals with IED and underscore the value of large-scale biobank data in clarifying health risks associated with psychiatric disorders.

## 1 Introduction

Intermittent Explosive Disorder (IED) is characterized by recurrent episodes of impulsive aggression that are disproportionate to situational stressors and often result in verbal or physical altercations, property destruction, or injury to others. With a lifetime prevalence of approximately 4–7% in community samples, IED represents a significant but frequently underdiagnosed condition with substantial personal, social, and public health implications (1, 2). Individuals with IED frequently experience comorbid psychiatric disorders, including mood, anxiety, and substance use disorders (3), as well as elevated rates of functional impairment and medical comorbidity (4). Despite its prevalence and clinical importance, health-related vulnerabilities beyond aggression remain insufficiently understood.

A growing body of evidence suggests that individuals with IED may be particularly vulnerable to adverse health behaviors and conditions, notably tobacco use and chronic pain. Epidemiological studies have consistently identified higher rates of nicotine dependence among individuals with IED compared to the general population (5). Shima et al. (6) reported a robust association between impulsive aggression, IED, and smoking behaviors, mirroring findings from broader research indicating that individuals with externalizing disorders are at heightened risk for substance-related problems (7). Tobacco use may function as a maladaptive coping mechanism for managing emotional dysregulation and interpersonal stress (8), while simultaneously lowering inhibition and exacerbating aggressive tendencies (5).

Emerging research also suggests a link between pain and externalizing psychopathology, including aggression. Chronic pain contributes to emotional dysregulation (9), irritability, and heightened stress responses (10)—factors known to increase the risk of aggressive behavior (11). Conversely, individuals with IED may exhibit heightened physiological arousal and stress reactivity that amplify pain perception. Bruehl et al. (12) noted that individuals with chronic pain display greater emotional reactivity and difficulties with anger regulation, potentially increasing their susceptibility to aggression. Although the overlap between pain and IED has not been extensively characterized, studies of externalizing disorders (13) indicate that pain complaints are common in these populations.

Despite these documented associations, clinical evaluations of individuals with IED often focus narrowly on aggression management, with limited attention to concurrent health vulnerabilities such as tobacco use and pain. Neglecting these domains may hinder comprehensive treatment, as unmanaged smoking and pain can reinforce aggressive behavior and worsen long-term outcomes.

However, much of the existing evidence linking IED to substance use and pain derives from small clinical samples or self-report surveys lacking population-level representation. Few studies have examined these associations within a large, diverse cohort while accounting for sociodemographic and psychiatric confounders. The current study extends prior research by leveraging the All of Us Research Program (14)—one of the largest U.S. population-based cohorts—to provide a more generalizable assessment of these relationships using rigorous propensity-score matching and covariate adjustment for comorbid major depressive disorder (MDD) and generalized anxiety disorder (GAD).

The present study leverages data from the All of Us Research Program (14), a large, diverse, population-based cohort, to investigate health-related vulnerabilities in individuals with and without IED. Specifically, we aimed to (1) examine the odds of tobacco use behaviors in individuals with IED compared to those without IED, and (2) evaluate whether individuals with IED report higher levels of pain. Based on prior literature linking impulsivity and externalizing psychopathology with smoking and pain sensitivity, we hypothesized that individuals with IED would exhibit elevated odds of tobacco use and greater pain severity relative to matched controls. By identifying patterns of increased vulnerability in these domains, this study seeks to inform clinical practice and underscore the importance of integrated behavioral health approaches extending beyond aggression management in IED.

## 2 Methods

### 2.1 Study design and participants

This study utilized data from the All of Us Research Program version 8, a large-scale, population-based cohort designed to advance precision medicine research and improve health outcomes across diverse populations in the United States (14) (see **Table 1**). Participants provided informed consent to share their health data, and the study adhered to the principles outlined in the Declaration of Helsinki. The present analysis focused on two groups: individuals who were diagnosed with a lifetime history of IED based on ICD codes extracted from electronic health records and a propensity matched comparison group without IED. Participants were included if they had completed survey assessments on substance use, pain, and social connectedness.

**Table 1:** Participant demographics.

Variable	Propensity-matched comparison group	IED	Effect size (type)
Total Participants	3,680	368	–
Female (%)	32.15	29.89	1.11 (OR)
Mean Age	52.20	52.69	0.04 (Cohen’s *d*)
White (%)	45.08	48.10	1.80 (OR)
Hispanic (%)	15.92	14.95	1.15 (OR)
Married (%)	30.49	27.17	0.99 (OR)
Unemployed (%)	47.01	67.66	2.54 (OR)

Demographic characteristics of the matched samples from the Propensity-Matched Comparison Group (PMCG) and the Intermittent Explosive Disorder (IED) group. Values represent percentages within each group or means for continuous variables. Effect sizes are expressed as Cohen’s d for continuous measures (e.g., Age) and odds ratios (ORs) for binary categorical variables (e.g., Female, White, Hispanic, Married, Unemployed). Cohen’s d values of approximately 0.2, 0.5, and ≥0.8 correspond to small, medium, and large effects, respectively. Odds ratios above 1 indicate greater odds in the IED group relative to the comparison group. Most demographic characteristics were well balanced following 10:1 propensity-score matching, though individuals with IED had substantially higher odds of unemployment (OR = 2.54).

### 2.2 Measures

Key variables were derived from participant responses to standardized survey items administered through the All of Us Research Program (14). Substance use was assessed through self-reported tobacco, alcohol, and other substance-related behaviors. Tobacco-related items included questions about lifetime cigarette use, current smoking frequency, electronic cigarette use, cigar smoking, and smokeless tobacco consumption. Alcohol use was measured based on self-reported drinking frequency in the past year and the number of drinks consumed on a typical day. Binge drinking behavior, defined as consuming six or more alcoholic beverages on a single occasion, was also recorded. Pain severity was assessed using a self-reported measure of average pain over the past seven days, with responses provided on a numeric scale ranging from 0 (no pain) to 10 (worst possible pain). Social isolation and companionship were measured through questions about the frequency of feeling a lack of companionship and the extent to which individuals felt they had someone to turn to for help when needed. Educational attainment and annual household income were obtained from self-reported demographic surveys. Additionally, the presence of comorbid MDD and GAD was identified using ICD diagnostic codes to account for potential psychiatric comorbidity effects.

### 2.3 Propensity score matching

**Figure 1:**
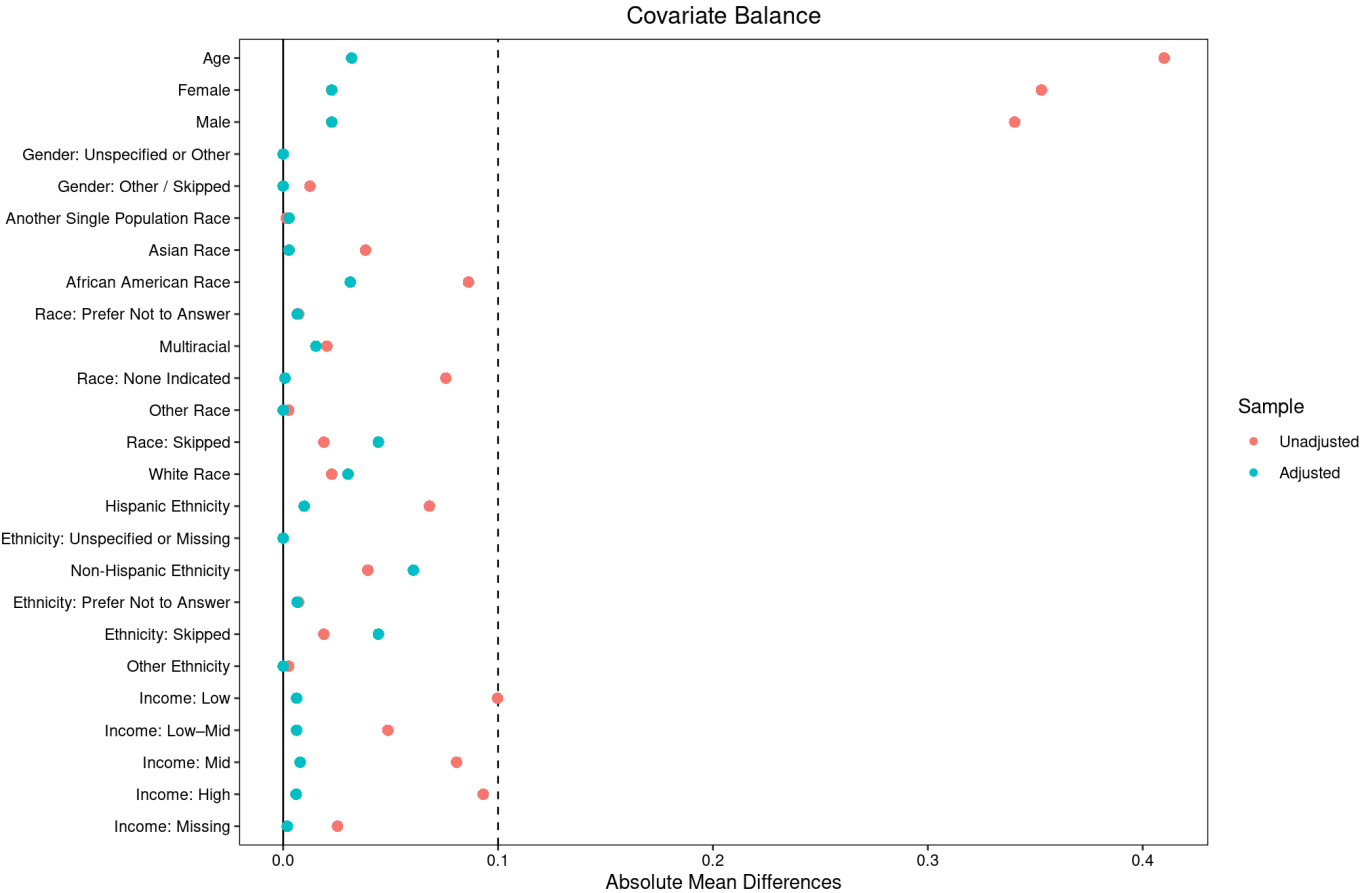
Covariate balance before and after matching in individuals with intermittent explosive disorder (IED) and the propensity matched comparison group. This plot displays standardized mean differences for demographic, socioeconomic, and clinical covariates before (unadjusted) and after (adjusted) 10:1 propensity-score matching in individuals with Intermittent Explosive Disorder (IED) compared with the matched comparison group. Covariates include age, sex, race, ethnicity, and income. The vertical dashed lines represent standardized mean-difference thresholds of ±0.1, commonly interpreted as indicating good covariate balance. Propensity-score matching markedly improved balance across all covariates, enhancing comparability between groups for subsequent analyses.

To reduce confounding and improve comparability between the IED and non-IED groups, a 10:1 propensity score matching procedure was employed (15). Propensity scores were estimated using logistic regression with the following covariates included as predictors of IED status: age (continuous), sex (male, female, other), race (White, Black, Asian, Other), ethnicity (Hispanic, Non-Hispanic), education level, household income, and the presence of MDD or GAD diagnoses. Nearest-neighbor matching without replacement was applied to match each individual with IED to up to ten individuals from the non-IED group based on their estimated propensity scores. This matching ratio was chosen to maximize statistical power while maintaining comparability between groups. Balance diagnostics were assessed following matching to ensure the adequate alignment of demographic characteristics across the two groups (see **Figure 1**).

### 2.4 Statistical analysis

Descriptive statistics were calculated to summarize demographic characteristics and survey responses for the IED and propensity matched comparison groups. Between-group comparisons were performed using chi-square tests (16) for categorical variables and t-tests for continuous variables. Logistic regression models and Fisher’s exact tests (16) were used to estimate odds ratios (ORs) (17) with 95% confidence intervals (CIs) for the association between IED status and key outcomes related to substance use, pain, and social isolation, adjusting for psychiatric co-morbidity of MDD and GAD. Income and education were not included as covariates in these outcome models because income was addressed through propensity matching and education exhibited high missingness. Odds ratios greater than 1 reflected an increased likelihood of the outcome among individuals with IED compared to the propensity-matched comparison group. To contextualize the magnitude of observed effects, we interpret ORs using established benchmarks: small effects correspond to ORs around 1.5, moderate effects to ORs around 2.5, and large effects to ORs of 4.0 or greater (18). To account for multiple comparisons and control the false discovery rate, p-values were adjusted using the Benjamini-Hochberg correction (19). This approach reduces the likelihood of false-positive findings while preserving statistical power in identifying significant associations. Statistical significance was defined as p < 0.05 after correction.

To enhance interpretability and reduce the influence of small cell counts and extreme odds ratio estimates, responses of “Prefer Not to Answer” and “Skip” were excluded only from the analyses of the specific variables where these responses were provided. To assess potential nonresponse bias, we examined differences in “Prefer Not to Answer” and “Skip” responses between the IED and propensity matched comparison groups across key survey items. While raw p-values suggested trends toward higher nonresponse in IED participants for alcohol and smoking questions, these differences were not statistically significant after Benjamini-Hochberg correction. Thus, nonresponse did not systematically differ between groups, reducing concerns about bias in reported results.

All statistical analyses were conducted using R statistical software (version 4.3.1) (20). Data processing and management were performed using the *dplyr* package (21). Propensity score matching was conducted utilizing the *MatchIt* package (22). Statistical analyses, including hypothesis testing, were carried out with functions from the base *stats* package.

## 3 Results

### 3.1 Sample description

The analytic sample included 368 individuals with IED and 3,680 matched controls from the All of Us Research Program (14). Following 10:1 propensity-score matching on age, sex, race/ethnicity, and income, demographic characteristics were well balanced across groups (**Table 1** and **Figure 1**). Participants were middle-aged on average (mean age ≈ 52 years), with approximately 30% identifying as female, 46–48% as White, and 15% as Hispanic or Latino. Unemployment rates remained higher in the IED group (68%) compared to the matched controls (47%). Within the broader All of Us dataset, IED diagnoses were relatively uncommon, representing approximately 0.1% of the total cohort.

### 3.2 Tobacco use

Individuals with IED demonstrated significantly higher odds of tobacco use compared to the propensity-matched comparison group. Specifically, they were more likely to have smoked at least 100 cigarettes in their lifetime (OR = 1.56, 95% CI = 1.25–1.94, adjusted p = 0.003). This finding indicates elevated likelihood of sustained tobacco exposure among individuals with IED.

### 3.3 Pain and physical health

Participants with IED also reported greater physical discomfort. They had higher odds of experiencing severe pain in the past seven days (7–10 on a 10-point scale) relative to matched controls (OR = 1.56, 95% CI = 1.22–1.99, adjusted p = 0.01). This pattern suggests that individuals with IED experience heightened physical pain burden alongside behavioral risk factors.

## 4 Discussion

Findings from the All of Us dataset align with and extend prior research indicating that individuals with IED exhibit greater vulnerability to adverse health behaviors and physical symptoms beyond aggression. Consistent with earlier epidemiologic and clinical studies (5, 6, 12, 23–25), individuals with IED in our sample showed significantly higher odds of tobacco use and greater self-reported pain severity compared to a propensity-matched comparison group. These results reinforce the view that IED is associated with broader dysregulation across behavioral and physiological domains, emphasizing the need for comprehensive assessment that includes both psychiatric and physical health factors.

### 4.1 Prevalence and context

The observed prevalence of IED within the All of Us cohort (approximately 0.1%) is notably lower than estimates from community-based epidemiological studies, which typically range from 4% to 7% (1). This discrepancy likely reflects the conservative diagnostic capture inherent to electronic health record (EHR)–based datasets, where IED is often underdiagnosed or misclassified under broader behavioral or mood disorder categories. Furthermore, All of Us participants represent individuals actively engaged in healthcare systems, who may differ demographically and clinically from the general population. Nevertheless, the pattern of associations identified here—particularly elevated tobacco use and pain burden among individuals with IED—aligns with prior research from community and clinical samples (2, 3, 26). supporting the external validity of these findings despite the lower absolute prevalence rate.

### 4.2 Tobacco use

The present findings corroborate well-established evidence that individuals with IED are more likely to engage in nicotine use and dependence than those without the disorder (5, 6). Epidemiological data have shown elevated rates of cigarette smoking, early initiation, and polysubstance use among individuals with impulsive aggression. Shima et al. (6) found that impulsive aggression and IED were strongly linked to tobacco and other substance use, while Scott et al. (3) reported nearly double the lifetime prevalence of alcohol abuse and dependence among those with IED. Our observation of higher odds of lifetime smoking, earlier smoking initiation, and heavier daily cigarette consumption in the All of Us cohort is consistent with these reports. Nicotine may serve as a maladaptive means of regulating irritability and arousal but may simultaneously heighten physiological activation and impulsivity (5), thereby perpetuating aggressive tendencies. These findings underscore the clinical importance of routine screening for tobacco use and offering smoking-cessation interventions as integral components of IED management.

### 4.3 Pain and physical health

Individuals with IED also demonstrated higher odds of reporting moderate-to-severe pain during the previous seven days, consistent with prior work linking pain conditions to externalizing and aggression-related psychopathology (12, 13). Bruehl et al. (12) proposed that individuals with chronic pain exhibit heightened emotional reactivity and difficulty regulating anger, which may increase susceptibility to aggressive outbursts. Conversely, chronic pain can contribute to irritability and stress-sensitivity, factors closely tied to the pathophysiology of IED (9–11). The bidirectional relationship between pain and aggression implies that each may exacerbate the other through shared mechanisms of dysregulated stress response and negative affect. Clinically, these results highlight the importance of evaluating pain symptoms in individuals presenting with impulsive aggression and considering integrated behavioral and medical interventions that address both pain and emotional regulation.

### 4.4 Clinical implications and future directions

Together, these findings point to two central health vulnerabilities in IED—tobacco use and pain—that may interact with emotional dysregulation to worsen overall functioning. Incorporating tobacco-cessation support, stress-management strategies, and multidisciplinary pain treatment into routine psychiatric care could enhance outcomes for individuals with IED. Future longitudinal and mechanistic studies should clarify causal pathways linking aggression, nicotine dependence, and pain perception, and test whether interventions addressing these domains can reduce both physical and behavioral morbidity.

### 4.5 Limitations

This study has several limitations. The cross-sectional design precludes causal inference. All behavioral and pain data were self-reported, raising potential recall and social-desirability bias. While the All of Us dataset is demographically diverse, individuals with more severe psychiatric illness may remain underrepresented. The comparison group excluded IED but not all other psychiatric conditions; however, major depressive disorder and generalized anxiety disorder were statistically controlled for. Education data had high missingness, limiting adjustment for socioeconomic differences, and unmeasured factors such as healthcare access may influence observed associations.

### 4.6 Conclusion

In summary, individuals with IED demonstrated higher odds of tobacco use and greater pain severity compared with matched controls, reflecting interrelated behavioral and somatic vulnerabilities. These results emphasize the need for integrated clinical approaches that extend beyond aggression management to include systematic screening and treatment for nicotine dependence and pain in individuals with IED.

## Data Availability

The datasets presented in this article are not readily available because this study leveraged data from the All of Us Research Program (https://allofus.nih.gov), funded by the National Institutes of Health. The analyzed data are available to authorized researchers through the All of Us Research Hub, following the program’s data access policies. Requests to access the datasets should be directed to support@researchallofus.org. Further inquiries can be directed to the corresponding author(s).
